# Symptom burden and secondary prevention in patients with left ventricular systolic dysfunction after acute myocardial infarction: a nationwide register-based study in Sweden

**DOI:** 10.1136/openhrt-2025-003506

**Published:** 2026-01-29

**Authors:** Eleonora Hamilton, Tomas Jernberg, Joakim Alfredsson, Christina Christersson, David Erlinge, Krister Lindmark, Elmir Omerovic, Liyew Desta, Christian Reitan

**Affiliations:** 1 Department of Clinical Sciences, Karolinska Institutet Institutionen för kliniska vetenskaper Danderyds sjukhus, Stockholm, Sweden; 2 Karolinska University Hospital, Stockholm, Sweden; 3 Linköping University, Linköping, Sweden; 4 Department of Medical Sciences, Cardiology, Uppsala University, Uppsala, Sweden; 5 Lund University, Lund, Sweden; 6 Department of Cardiology, Sahlgrenska University Hospital, Gothenburg, Sweden; 7 Department of Medicine, Karolinska Institutet, Stockholm, Sweden

**Keywords:** HEART FAILURE, Coronary Artery Disease, Risk Factors, Epidemiology, Acute Coronary Syndrome

## Abstract

**Background:**

There is a lack of contemporary data describing patients with left ventricular (LV) systolic dysfunction post myocardial infarction (MI) in terms of symptom burden and secondary prevention measures. The aim of this study was to describe patients with various degrees of LV systolic dysfunction after a first MI, their symptom burden, quality of life and adherence to recommended secondary prevention measures in a nationwide patient material.

**Methods:**

Patients (n=49 564) registered in the Swedish Web-System for Enhancement and Development of Evidence-Based Care in Heart Disease registry between 2011 and 2018, diagnosed with a first acute MI, discharged alive and with no previous heart failure, were stratified by degree of LV systolic dysfunction.

**Results:**

Compared with patients with normal ejection fraction (EF≥50%), patients with a reduced EF (<30%) more often experienced shortness of breath (32.3% vs 5.6%, adjusted OR (95% CI): 7.45 (6.22 to 8.92)), had more often been readmitted (48.1% vs 31.2%, 1.87 (1.61 to 2.19)) and were more often on sick leave (26.6% vs 9.5%, 3.35 (2.45 to 4.58)), whereas there were no significant differences regarding chest pain and quality of life at the follow-up visit after 11–13 months. Patients with EF <30% had participated in education programme (44.9% vs 55.5%, 0.70 (0.60 to 0.81)) and physical therapy (11.3% vs 14.9%, 0.68 (0.58 to 0.79)) and have been physically active at least 30 min per day for at least 5 days per week (35.5% vs 40.2%, 0.86 (0.73 to 1.01)) to a lesser extent.

**Conclusion:**

Contemporary representative data show that LV systolic dysfunction after MI is associated with a very high symptom burden and worse secondary prevention after 11–13 months.

**WHAT IS ALREADY KNOWN ON THIS TOPIC**

- Patients with left ventricular systolic dysfunction (LVSD) following acute myocardial infarction (AMI) are at increased risk of adverse outcomes. While pharmacological treatment is well-studied, there is limited knowledge about patients’ symptom burden and their participation in secondary prevention programmes within routine care.

**WHAT THIS STUDY ADDS**

- This nationwide study provides real-world data showing that many patients with LVSD experience substantial symptom burden and limited participation in secondary prevention activities 1 year after AMI. The study also highlights disparities between patient groups, including sex differences in care and follow-up.

**HOW THIS STUDY MIGHT AFFECT RESEARCH, PRACTICE OR POLICY**

- The findings underscore the need for structured, equitable follow-up after AMI, with greater focus on symptom relief and patient-centred secondary prevention. This may help guide improvements in long-term care and support for patients with systolic dysfunction.

## Introduction

 Despite the prominent advances in the treatment of coronary artery disease in the last decades, acute myocardial infarction (MI) remains the most common cause of heart failure (HF).^1 2^ Development of HF after MI is associated with adverse events, impaired quality of life and higher mortality.^3 4 4^ There has been significant progress in the treatment of HF during the last 10–15 years. The contemporary risks and trends in the development of HF after an index MI have recently been studied and reported.^5^ However, there is a lack of contemporary representative data describing outcome in terms of symptoms, quality of life and adoption of secondary prevention in patients with LV systolic dysfunction after MI.

Sweden is one of the few countries in the world with a continuous national quality of care and outcome registry for acute MI in which all hospitals participate,^6^ and therefore has an optimal setting to perform nationwide epidemiological studies. The Swedish Web-System for Enhancement and Development of Evidence-Based Care in Heart Disease (SWEDEHEART) registry collects detailed data on the in-hospital course as well as during follow-up. It records symptoms, quality of life using EuroQol-5 Dimensions (EQ-5D), as well as secondary prevention measures after 3 months and 12 months post MI.

The purpose of this study was to describe patients’ symptom burden, quality of life and adherence to recommended secondary prevention measures in relation to LV ejection fraction (EF) in a nationwide cohort of patients surviving the first year after a first MI.

## Methods

### Study population

Data were obtained from the SWEDEHEART registry. The registry is an established national quality register including all Swedish hospitals that provide acute cardiac care (n=72) and details about the registry can be found elsewhere.^6^ Nearly all patients in Sweden admitted to hospitals with an acute MI are enrolled continuously. All patients are informed about the registry and their right to opt out. The study population included male and female adults ≥18 years with no previous HF, registered in SWEDEHEART between 2011 and 2018, diagnosed with a first-time acute spontaneous (type 1) MI (according to the European Society of Cardiology, American College of Cardiology and American Heart Association consensus documents,^6^ discharged alive and with no coronary artery bypass graft surgery (CABG) during hospitalisation. Patients >74 years of age before 2018 and patients >79 years of age from 2018 and onward were excluded, since they were not eligible for secondary prevention follow-up in SWEDEHEART and could therefore not be assessed for 1-year outcomes. In addition, patients without echocardiographic assessment during the index hospitalisation were excluded, as LVEF was a central variable for stratifying patients by degree of systolic dysfunction and for evaluating associations with symptom burden. The population was divided into four subgroups defined by degree of LV systolic function: EF≥50%, EF 40–49%, EF 30–39% and <30%, according to how it is registered in the SWEDEHEART registry. Patients were then assessed 11–13 months after MI.

### Clinical characteristics and diagnoses

Baseline characteristics, in-hospital course data (including examinations, interventions and complications), discharge medications and diagnoses were obtained from SWEDEHEART. The study database was also enriched with data from the National Patient Registry (NPR) regarding the history of diabetes mellitus (International Classification of Diseases (ICD) code: E10-14, 250), renal failure (ICD codes: Z49, V56A, V45B, Z992), MI (ICD codes: I21-23, 410, 412), stroke (ICD codes: I60-64, 430–36), peripheral artery disease (ICD codes: I70-73, 440–43), chronic pulmonary disease (ICD codes: J40-47, 491–496), dementia (ICD codes: G30-31, 290, 294B) and cancer diagnosis (ICD codes: C14 - C20) within 3 years. The NPR includes discharge diagnoses for all patients hospitalised in Sweden since 1987. To calculate the estimated glomerular filtrate rate (eGFR), the Chronic Kidney Disease Epidemiology Collaboration equation was used.^7 8^ eGFR was calculated in mL/min/1.73 m^2^ and dichotomised at <60 mL/min/1.73 m^2^ (normal/impaired renal function).

### Outcomes

Endpoints were perceived symptoms as well as secondary prevention measures evaluated 11–13 months after discharge, according to follow-up visits in SWEDEHEART. Symptoms included dyspnoea (severity classified according to the New York Heart Association (NYHA)) and chest pain (severity classified according to the Canadian Cardiovascular Society (CCS)). As measurements of symptom burden, we also measured readmissions, sick leave and quality of life (measured with EQ-5D). The EQ-5D descriptive system includes one question for each of the five dimensions: level of mobility, hygiene/self-care, level of activities, pain/discomfort and anxiety/depression. The median EQ-5D of all patients was calculated and dichotomised (<0.85 or not). Secondary prevention measures included participation in the cardiac rehabilitation education programme, physiotherapy programme, daily physical activity >5 times a week, current smoking and target level of Low density lipoprotein-cholesterol (LDL).

Cardiac rehabilitation education programme is an education about lifestyle risk factors, smoking, exercise, diet, etc. Doctors and nurses are leading the course together with physiotherapists and dietitians. Physical therapy is defined as attendance in a structured exercise programme for at least 3 months during the first year post MI.

### Statistical analyses

Descriptive analyses were used for demographics and characteristics at baseline and at follow-up and are shown as numbers and proportions for categorical variables and median (IQR) or mean (SD) for continuous variables. Bar charts were used to illustrate the differences between the groups, stratified by EF, regarding symptoms, defined as dyspnoea (NYHA-class ≥2, chest pain (CCS-class ≥2), quality of life (EQ-5D) (<0.85), readmission to hospital and sick leave, as well as secondary prevention measures including education programme, physical therapy participation at least 3 months, physical activity (≥30 min, ≥5 days a week), current smoking and LDL-cholesterol (<1.4 mmol/L). In the multivariable logistic regression analyses, we then adjusted for age, sex, body mass index, smoking, hypertension, hyperlipidaemia, diabetes mellitus type 2, chronic kidney disease stage 3–5, previous Percutaneous Coronary Intervention (PCI), previous stroke, peripheral artery disease, Chronic obstructive pulmonary disease (COPD), dementia, cancer, dialysis and ST-elevation MI (STEMI).

## Results

### Study population

**Figure 1:**
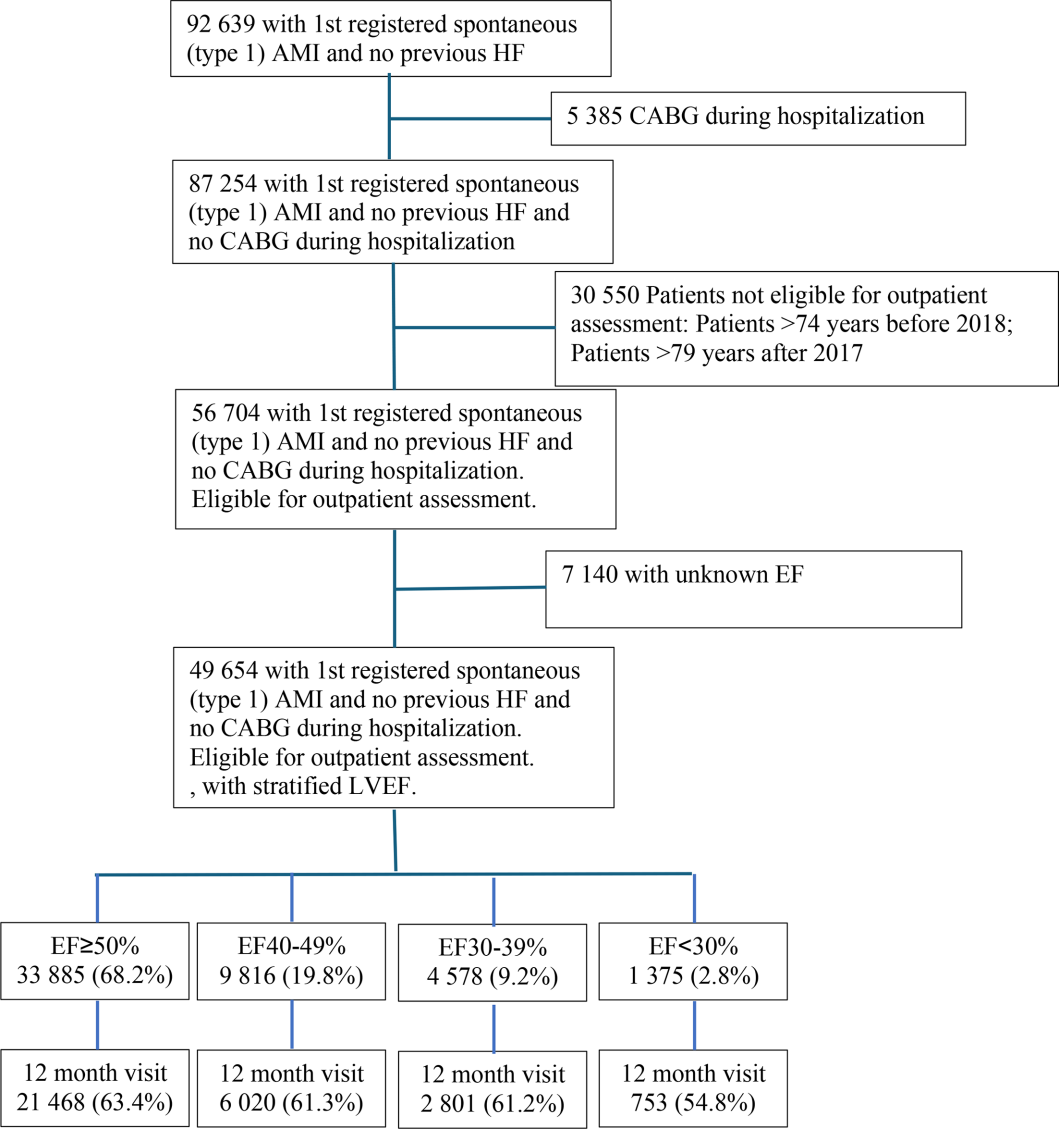
Flow chart. AMI, acute myocardial infarction; CABG, coronary artery bypass graft surgery; EF, ejection fraction; HF, heart failure; LV, left ventricular.

A total of 92 639 patients without previously known HF were discharged alive after a first-time spontaneous (type 1) acute MI between 1 January 2011 and 20 May 2018, of which 87 254 did not undergo CABG during hospitalisation. 30 550 were not eligible for outpatient assessment in SWEDEHEART (age over 74 years until 2018, age over 79 years thereafter). Out of the remaining 56 704, there were 7140 who had no EF estimated at index hospitalisation, leaving a total of 49 654 to be included in the present analyses (figure 1). There were 33 885 (68.2%) with normal EF (≥50%), 9816 (19.8%) with slightly impaired EF (40–49%), 4578 (9.2%) with moderately impaired EF (30–39%) and 1375 (2.8%) with severely impaired EF (<30%).

### Baseline characteristics

When comparing patients with different EF, those with lower EF were somewhat older and more often male (table 1). Going from normal EF to severely impaired EF, the proportion of patients being a current smoker, having diabetes mellitus and chronic kidney disease increased. Patients with severely impaired EF presented more often with STEMI, had more often atrial fibrillation and received more often intravenous diuretics compared with those with normal EF. As expected, patients with impaired EF had more often ACE inhibitor, beta-blockers and aldosterone receptor antagonist at discharge compared with those with normal EF.

**Table 1:** . Baseline demographics and characteristics on admission and discharge

	Normal LVEF	Slightly impaired LVEF	Moderately impaired LVEF	Severely impaired LVEF	P value
	EF≥50%	EF 40–49%	EF 30–39%	EF<30%	
	(n=33 885)	(n=9816)	(n=4578)	(n=1375)	
Demography					
Age, mean (SD)	62 (9)	63 (9)	63 (8)	64 (8)	<0.001
Male, n (%)	24 794 (73)	7506 (77)	3450 (75)	1062 (77)	<0.001
Risk factors					
BMI, mean (SD)	28 (6)	28 (5)	28 (9)	27 (5)	0.001
Current smoker	9894 (30)	3146 (33)	1551 (35)	538 (41)	<0.001
Hypertension, n (%)	16 035 (47)	4505 (46)	2130 (47)	640 (47)	0.082
Hyperlipidaemia, n (%)	7677 (23)	2118 (22)	1015 (22)	324 (24)	0.098
Diabetes mellitus, n (%)	6476 (19)	2115 (22)	1165 (26)	433 (32)	<0.001
eGFR (mean (SD))	85 (18)	83 (19)	82 (20)	78 (22)	<0.001
CKD stage 3, n (%)	2996 (9)	1119 (12)	624 (14)	253 (19)	<0.001
Previous cardiovascular					
PCI, n (%)	3673 (11)	965 (10)	398 (9)	131(10)	<0.001
CABG, n (%)	1249 (4)	436 (4)	182 (4)	62 (5)	0.004
Stroke, n (%)	1200 (4)	384 (4)	244 (5)	74 (5)	<0.001
Peripheral artery disease, n (%)	789 (2)	271 (3)	180 (4)	66 (5)	<0.001
Previous other disease					
COPD*, n (%)	1409 (4)	466 (5)	255 (6)	116 (8)	<0.001
Dementia, n (%)	48 (0)	15 (0)	6 (0)	2 (0)	0.990
Cancer, n (%)	549 (2)	203 (2)	94 (2)	27 (2)	0.008
Presentation					
Cardiopulmonary resuscitation before hospital, n (%)	514 (2)	318 (3)	232 (5)	75 (6)	<0.001
STEMI, n (%)	11 911 (35)	5810 (60)	2992 (65)	828 (60)	<0.001
Sinus, n (%)	32 360 (96)	9164 (94)	4184 (92)	1172 (86)	<0.001
Atrial fibrillation/flutter, n (%)	939 (3)	401 (4)	245 (5)	137 (10)	<0.001
Systolic blood pressure (median (IQR))	153 (28)	148 (29)	144 (29)	138 (31)	0.241
Reperfusion in STEMI					
Acute coronary angiogram without reperfusion, n (%)	272 (3)	99 (2)	64 (2)	30 (4)	0.002
Primary PCI†, n (%)	10 313 (95)	5013 (95)	2498 (94)	645 (92)	0.002
Trombolysis, n (%)	313 (3)	179 (3)	85 (3)	25 (4)	0.002
Treatment					
PCI† during hospital stay, n (%)	29 058 (86)	8868 (90)	4028 (88)	1140 (83)	<0.001
Intravenous diuretics, n (%)	1440 (4)	1383 (14)	1369 (30)	752 (55)	<0.001
Medication at discharge					
Aspirin, n (%)	32 916 (97)	9443 (96)	4337 (95)	1248 (91)	<0.001
Statins, n (%)	33 013 (97)	9549 (97)	4423 (97)	1286 (94)	<0.001
Beta-blockers, n (%)	29 835 (88)	9145 (93)	4383 (96)	1322 (96)	0.003
ACEI, n (%)	21 052 (62)	7422 (76)	3596 (79)	1068 (78)	0.005
ARB‡, n (%)	6426 (19)	1733 (18)	784 (17)	238 (17)	<0.001
ACEI or ARB‡, n (%)	27 253 (80)	9088 (93)	4355 (95)	1296 (94)	0.003
Diuretics, n (%)	3376 (10)	1426 (15)	1263 (28)	700 (51)	<0.001
MRA, n (%)	602 (2)	600 (6)	821 (18)	449 (33)	<0.001
Oral Anticoagulatia, n (%)	574 (2)	282 (3)	182 (4)	72 (5)	<0.001
Attended follow-up					
Attended second follow-up, n (%)	21 468 (63)	6020 (61)	2801 (61)	753 (55)	<0.001
Occupation status					
Employed, n (%)	14 593 (43)	3764 (38)	1614 (35)	413 (30)	<0.001
Sick leave, n (%)	1102 (3)	298 (3)	124 (3)	46 (3)	<0.001
Unemployed, n (%)	792 (2)	249 (3)	116 (3)	29 (2)	<0.001
Retired, n (%)	15 124 (45)	4760 (49)	2416 (53)	763 (56)	<0.001
Studies/other, n (%)	244 (1)	80 (1)	27 (1)	14 (1)	<0.001

* Chronic obstructive pulmonary disease

† Percutaneous Coronary Intervention

‡ Angiotensin II receptor blocker

ACEI, ACE inhibitor; BMI, body mass index; CABG, coronary artery bypass graft surgery; CKD, chronic kidney disease; EF, ejection fraction; eGFR, estimated glomerular filtrate rate; LV, left ventricular; MRA, aldosterone receptor antagonist; STEMI, ST-elevation myocardial infarction.

### Attendance of follow-up visit after 11–13 months

Out of 49 654 discharged patients, 1253 (0.9%) died and 17 359 (23.4%) did not attend for other reasons, leaving 31 042 patients who attended the 11–13 months follow-up visit. The attendance decreased with decreasing EF (figure 1, table 1).

### Symptoms

**Figure 2:**
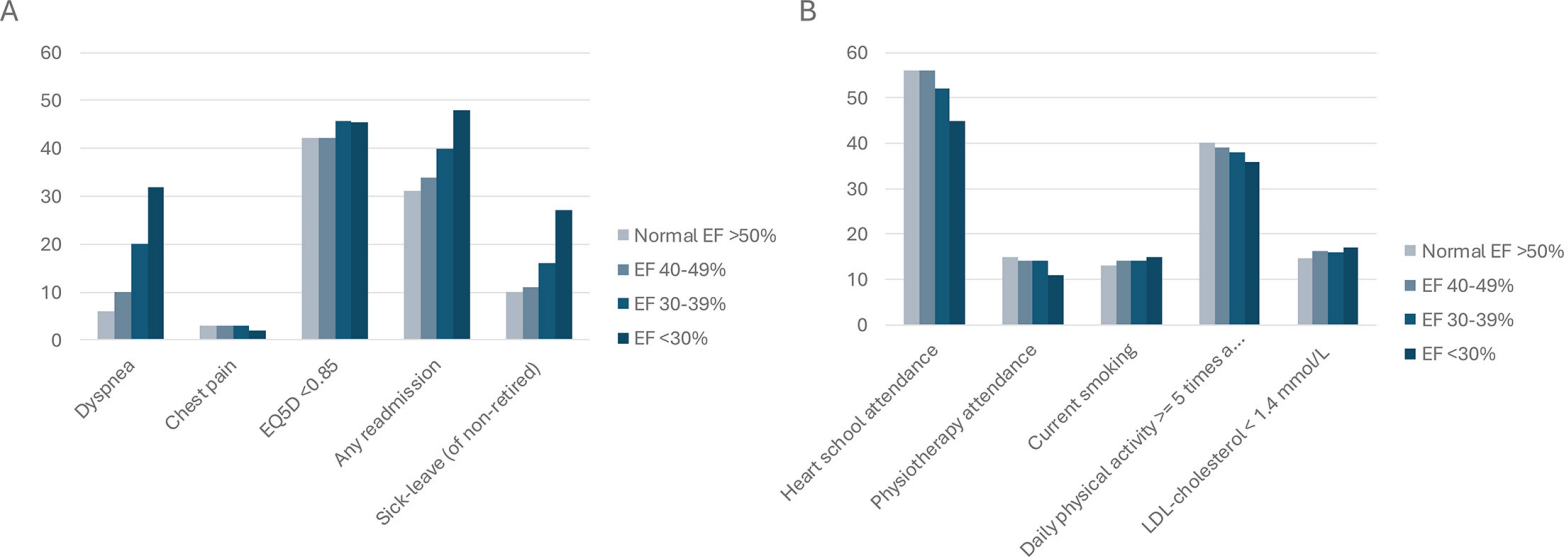
(**A**) Dyspnoea, chest pain, quality of life (EQ-5D), readmissions and sick leave in relation to ejection fraction (EF). (**B**) Heart school attendance, physiotherapy attendance, daily physical activity ≥5 times a week, LDL-cholesterol <1.4, and current smoking in relation to EF. EQ-5D, EuroQol-5 Dimensions.

1 year after the index MI, patients with impaired EF more often experienced dyspnoea (32% for EF <30 vs 6% for EF ≥50) (table 2, figure 2A). There was no major difference in perceived chest pain between the different groups. Patients with EF <30% tended to have more difficulties regarding all five dimensions of EQ-5D (table 3) and had a somewhat lower mean EQ-5D score than patients with normal EF (0.80 vs 0.83). The proportion with reduced EQ-5D (<0.85) was higher in patients with EF <30% than in patients with normal EF (45% vs 42%). The risk of any readmission increased with decreasing EF. For non-retired, the proportion of sick leave increased with decreasing EF. In the adjusted analyses (table 4), a lower EF was still significantly associated with dyspnoea, need for readmission and sick leave, but not with chest pain and reduced quality of life.

**Table 2:** Prevalence of symptoms and functional status 12 months after myocardial infarction

	12 months post MI				P value
	EF ≥50%	EF 40–49%	EF 30–39%	EF <30%	
	n=21 468	n=6020	n=2801	n=753	
Symptoms					
Dyspnoea, n (%)	1200 (6)	614 (10)	550 (20)	241 (32)	<0.001
Non-cardiac chest pain, n (%)	802 (4)	213 (4)	74 (3)	12 (2)	0.003
CCS II-IV, n (%)	618 (3)	171 (3)	87 (3)	15 (2)	0.003
EQ-5D (mean (SD)), n (%)	0.83 (0.23)	0.83 (0.23)	0.81 (0.25)	0.80 (0.25)	<0.001
EQ-5D < 0.85, n (%)	9010 (43)	2515 (42)	1272 (46)	339 (45)	0.002
Any readmission, n (%)	6776 (31)	2110 (34)	1137 (40)	376 (48)	<0.001
Sick leave (of non-retired), n (%)	984 (10)	|290 (11)	|176 (16)	|69 (27)	<0.001
Prevention measures					
Heart school attendance (%)	12 924(56)	3662 (56)	1566 (52)	358 (45)	<0.001
Physiotherapy attendance: yes, <3 months, n (%)	8996 (39)	2560 (40)	1082 (36)	249 (32)	<0.001
Physiotherapy attendance: yes, ≥3 months, n (%)	3423 (15)	907 (14)	421 (14)	89 (11)	<0.001
Physical activity, 30 min a day (%)					
0 days a week, n (%)	4416 (21)	1346 (23)	729 (26)	226 (30)	<0.001
2–4 days a week, n (%)	7259 (34)	2083 (33)	875 (31)	213 (29)	<0.001
≥5 days a week, n (%)	8574 (40)	2345 (39)	1059 (38)	265 (36)	0.008
Current smoking	2778 (13)	830 (14)	383 (14)	114 (15)	0.003
LDL-cholesterol* <1.4	2871 (15)	886 (16)	410 (16)	113 (17)	0.013

* Low density lipoprotein (LDL)

CCS, Canadian Cardiovascular Society; EF, ejection fraction; EQ-5D, EuroQol-5 Dimensions; MI, myocardial infarction.

**Table 3:** EuroQol-5 Dimensions (EQ-5D) in relation to ejection fraction (EF)

	n=21 468	n=6020	n=2801	n=753	P value
	EF ≥50%	EF 40–49%	EF 30–39%	EF <30%	
Level of mobility, n (%)					
Walk without difficulty	17 949 (84)	4938 (83)	2220 (78)	535 (72)	<0.001
Walk with some difficulty	3307 (16)	1026 (17)	550 (20)	211 (28)	<0.001
Bedridden	59 (0)	16 (0)	16 (0)	1 (0)	<0.001
Level of hygiene, n (%)					
No need for help with daily hygiene, food and dressing	25 113 (98)	7181 (97)	3209 (96)	817 (94)	<0.001
Have some difficulty washing or dressing themselves	530 (2)	161 (2)	111 (3)	47 (5)	<0.001
Can not wash or dress themselves	50 (0)	27 (0)	16 (1)	8 (1)	<0.001
Level of activities, n (%)					
Manage their main activities	22 030 (86)	6168 (84)	2678 (80)	607 (70)	<0.001
Some difficulty in coping with main activity	3209 (13)	1027 (14)	554 (17)	221 (25)	<0.001
Unable to perform their main activities	450 (2)	173 (2)	103 (3)	43 (5)	<0.001
Level of pain, n (%)					
No pain or discomfort	15 859 (62)	4568 (62)	2053 (62)	459 (53)	0.179
Moderate pain or discomfort	8875 (35)	2538 (34)	1181 (35)	380 (44)	0.179
Severe pain or discomfort	947 (4)	260 (4)	99 (3)	33 (4)	0.179
Level of discomfort and sadness, n (%)					
No anxiety or depression	16 074 (63)	4630 (63)	2029 (61)	492 (56)	0.016
Moderate anxiety or depression	8552 (33)	2422 (32)	1177 (35)	347 (40)	0.016
Severe anxiety or depression	1047 (4)	314 (4)	127 (4)	33 (4)	0.016
Current health-related quality of life, scale from 1 to 100, median (IQR)	71 (20)	71 (19)	69 (19)	66 (19)	<0.001

**Table 4:** Adjusted analyses

EF 50% ref	EF 40–49%	EF 30–39%	EF <30%
	OR (95% CI)	OR (95% CI)	OR (95% CI)
Symptoms			
Dyspnoea	1.86 (1.66 to 2.08)	4.07 (3.61 to 4.60)	7.45 (6.22 to 8.92)
Chest pain	1.00 (0.83 to 1.21)	1.11 (0.87 to 1.42)	0.75 (0.45 to 1.28)
EQ-5D<0.85	1.01 (0.95 to 1.08)	1.16 (1.07 to 1.27)	1.11 (0.95 to 1.31)
Any readmission	1.14 (1.07 to 1.22)	1.37 (1.26 to 1.49)	1.87 (1.61 to 2.19)
Sick leave	1.11 (0.96 to 1.30)	1.67 (1.38 to 2.03)	3.35 (2.45 to 4.58)
Prevention measures			
Heart school	0.99 (0.93 to 1.05)	0.84 (0.78 to 0.91)	0.70 (0.60 to 0.81)
Physical therapy	0.98 (0.92 to 1.04)	0.86 (0.80 to 0.94)	0.68 (0.58 to 0.79)
Physical activity	0.93 (0.87 to 0.99)	0.90 (0.82 to 0.98)	0.86 (0.73 to 1.01)
Current smoking	1.04 (0.93 to 1.15)	0.89 (0.77 to 1.03)	0.78 (0.61 to 1.00)
LDL-cholesterol <1.4	1.06 (0.97 to 1.16)	1.05 (0.93 to 1.18)	1.04 (0.84 to 1.30)

OR for different outcomes per LVEF stratum, compared with normal LVEF (≥50%).

EF, ejection fraction; EQ-5D, EuroQol-5 Dimensions; LVEF, left ventricular ejection fraction.

### Secondary prevention

Patients with low EF participated in the education programme and the physiotherapy programme to a lesser extent and were less physically active than those with normal EF at the 1year follow-up. They were somewhat more often smokers and had more often LDL-cholesterol <1.4 mmol/L (table 2, figure 2B). In the adjusted analysis, a lower EF was still significantly associated with a low education programme and physiotherapy attendance, and low physical activity but not with smoking or LDL-cholesterol <1.4 mmol/L (table 4).

## Discussion

In this nationwide study of patients with a first-time MI without previous HF across an entire country, a reduced EF was strongly associated with a higher symptom burden and lower quality of life. Among those with the most severe LV systolic dysfunction (EF <30%), nearly half were readmitted to hospital within the first year. Approximately one-third reported symptoms of dyspnoea, and one-third remained on sick leave after 1 year. Patients with severe LV systolic dysfunction participated less frequently in education and physical therapy programmes, and they were less physically active after 1 year. These findings suggest that secondary prevention programmes may need to align better with the needs of patients with LV systolic dysfunction.

This is a large observational study, describing symptom burden, quality of life and adherence to recommended secondary prevention measures in a real-world cohort of post-MI patients with various degrees of LV systolic dysfunction. The SWEDEHEART registry is one of the few registries that continuously records the process of care for acute MI, with participation from all hospitals in the country.^6^ Sweden’s population, healthcare system and incidence of cardiovascular disease align with those of numerous developed nations, enhancing the study’s generalisability. It is imperative to recognise the importance of long-term health-related symptom observations, as they are often overlooked in both research and clinical practice. The primary focus tends to be on preventing recurrent cardiac events and death.

The mean age of the study sample, 62–64 years, was lower than seen in other studies including unselected MI patients.^9^ This is explained by the fact that the present study included only first-time MIs, and only individuals below 75 years of age until 2018 and below 80 years thereafter were included in the registry for the outcomes of interest. Moreover, the mean age decreased when patients who did not have an echocardiogram during their hospital stay were excluded.^9^ The proportion of men being around 75% is in line with the general MI population in Sweden.^9^ Patients with severely reduced EF (<30%) had a higher proportion of risk factors such as diabetes mellitus and chronic kidney disease and were smokers to a higher extent, which is also well in line with earlier studies.^5^ Of note, the amount of atrial fibrillation/flutter at presentation was significantly higher in those with severe systolic LV dysfunction (EF <30%) (10%), compared with the other groups (2–5%), but the amount of Oral anticoagulantia at discharge did not differ as much (2–5%), which might indicate that the most vulnerable groups are undertreated.

It is important to note that many patients did not attend the follow-up visit. Death during the first year was more common in those with lower EF. There was also a large proportion who did not attend follow-up for other reasons, and patients with reduced EF had the lowest attendance, suggesting a potential selection of the healthiest patients in the group with reduced EF. Hence, those with most symptoms, poorest quality of life and worst prognosis were less likely to attend follow-up visits. One possible explanation could be that the threshold for participating in these programmes may be too high for the patients with the most symptoms. Recent studies have shown that structured follow-up after MI can benefit patients’ quality of life.^10^,^12^ Thus, healthcare providers face significant challenges in reaching out to these vulnerable patients who have a higher risk of mortality.^5 13^

Despite this selection, there was still a strong association between EF, NYHA class, hospitalisations and sick leave, while the association with quality of life was weaker. Our findings align with many other studies on the relationship between the degree of HF, EF and the subsequent symptom burden and quality of life.^3 14^ The novelty and importance of our study lie not in the associations themselves, but rather in describing the symptom burden and the impact on quality of life in a contemporary and unselected population of post-MI patients.

The rate of sick leave at admission was very similar for all groups (3%). Of patients who were working or on sick leave on admission, those with EF <30% had the highest rate of sick leave (27%) after 1 year. The group with an EF of 30–39% had a sick leave rate of 16%, which was still higher than the other groups (10–11%). The overall rate of sick leave agrees with results from a recent study in employed patients with MI, where the sick leave rate was 41% at 60 days and 7% at 365 days.^15^

Most patients in the present study were offered cardiac rehabilitation. However, compliance with physical therapy was low across all groups, with the poorest compliance observed in the group with the worst EF. This is concerning, as previous studies affirm that cardiac rehabilitation is highly effective after a MI to reduce the risks of hospital readmissions, cardiac mortality and recurrent MI.^16^ Additionally, research shows that patients who remain active during follow-up periods after a MI tend to report higher EQ-5D scores.^17^

It is recommended that patients with chronic coronary syndrome engage in physical activity for 30–60 min, 5 days a week, as it has been shown to reduce mortality.^417^,^19^ However, the adherence to these recommendations varies greatly.^20^,^23^ Despite these recommendations, 20–30% of patients across all groups reported having no daily physical activity at follow-up. Only a small number of patients did meet the recommended levels of physical activity, and this was consistent across all groups. Only 36–40% of patients had physical activity at the recommended levels. There was no significant difference in physical activity levels between groups. A previous study has shown similar results where patients with normal LVEF were more likely to be both constantly inactive and constantly active.^17^

This study has some limitations. Although the validity of the registry is high, the data quality is not as high as in clinical trials or well-executed observational studies. Echocardiography was not performed in 7140 patients, who were excluded from the study. A larger proportion of patients with reduced EF was lost to follow-up because of death within 1 year or other reasons for not attending follow-up visits, most likely leading to an underestimation of the association between EF, symptom burden and quality of life. Because this is an observational study and residual confounding cannot be excluded, conclusions regarding causality should not be made. Another limitation is that several contemporary therapies and clinical characteristics relevant to symptom burden could not be fully evaluated. Angiotensin receptor-neprilysin inhibitors (ARNIs) are now a cornerstone of treatment for HF with reduced EF. However, their clinical use postdated much of the study period, and ARNI use was therefore very limited in this population. Similarly, sodium-glucose cotransporter 2 inhibitors, which are now recommended for patients with Heart faulire with reduced ejection fraction regardless of diabetes status, were not established therapies during the study period. We also lacked data on valvular heart disease. Although likely rare in this population, severe valvular disease may have influenced symptom burden in some patients. Finally, we did not have data on completeness of revascularisation. Although prior studies suggest only modest effects of complete revascularisation on symptoms,^24 25^ we could not account for the potential impact of residual ischaemia on the studied endpoints.

In conclusion, patients with reduced EF after MI had higher symptom burden and lower quality of life after 1 year. Of those with EF <30%, half were readmitted to the hospital within 1 year, one-third had dyspnoea and one-third were on sick leave after 1 year. Despite the poor prognosis, patients with reduced EF participated less frequently in heart school and physical therapy programmes, and they were less physically active after 1 year.

## Data Availability

Data may be obtained from a third party and are not publicly available.
